# A new anti-glioma therapy, AG119: pre-clinical assessment in a mouse GL261 glioma model

**DOI:** 10.1186/s12885-015-1538-9

**Published:** 2015-07-17

**Authors:** Rheal A. Towner, Michael Ihnat, Debra Saunders, Anja Bastian, Nataliya Smith, Roheeth Kumar Pavana, Aleem Gangjee

**Affiliations:** 1 Advanced Magnetic Resonance Center, Oklahoma Medical Research Foundation, Oklahoma City, OK 73104 USA; 2 Pharmaceutical Sciences, College of Pharmacy, University of Oklahoma Health Sciences Center, Oklahoma City, OK 73117 USA; 3 Department of Physiology, College of Medicine, University of Oklahoma Health Sciences Center, Oklahoma City, OK 73117 USA; 4 Graduate School of Pharmaceutical Sciences, Duquesne University, Pittsburgh, PA 15282 USA

**Keywords:** AG119, Anti-angiogenic, Anti-microtubule, Anti-cancer, GL261 mouse glioma, Magnetic resonance imaging (MRI), Methyl guanine transferase (MGMT), High-grade gliomas (HHGs), Temozolomide (TMZ)

## Abstract

**Background:**

High grade gliomas (HGGs; grades III and IV) are the most common primary brain tumors in adults, and their malignant nature ranks them fourth in incidence of cancer death. Standard treatment for glioblastomas (GBM), involving surgical resection followed by radiation and chemotherapy with temozolomide (TMZ) and the anti-angiogenic therapy bevacizumab, have not substantially improved overall survival. New therapeutic agents are desperately needed for this devastating disease. Here we study the potential therapeutic agent AG119 in a pre-clinical model for gliomas. AG119 possesses both anti-angiogenic (RTK inhibition) and antimicrotubule cytotoxic activity in a single molecule.

**Methods:**

GL261 glioma-bearing mice were either treated with AG119, anti-VEGF (vascular endothelial growth factor) antibody, anti c-Met antibody or TMZ, and compared to untreated tumor-bearing mice. Animal survival was assessed, and tumor volumes and vascular alterations were monitored with morphological magnetic resonance imaging (MRI) and perfusion-weighted imaging, respectively.

**Results:**

Percent survival of GL261 HGG-bearing mice treated with AG119 was significantly higher (*p* < 0.001) compared to untreated tumors. Tumor volumes (21–31 days following intracerebral implantation of GL261 cells) were found to be significantly lower for AG119 (*p* < 0.001), anti-VEGF (*p* < 0.05) and anti-c-Met (*p* < 0.001) antibody treatments, and TMZ-treated (*p* < 0.05) mice, compared to untreated controls. Perfusion data indicated that both AG119 and TMZ were able to reduce the effect of decreasing perfusion rates significantly (*p* < 0.05 for both), when compared to untreated tumors. It was also found that IC_50_ values for AG119 were much lower than those for TMZ in T98G and U251 cells.

**Conclusions:**

These data support further exploration of the anticancer activity AG119 in HGG, as this compound was able to increase animal survival and decrease tumor volumes in a mouse GL261 glioma model, and that AG119 is also not subject to methyl guanine transferase (MGMT) mediated resistance, as is the case with TMZ, indicating that AG119 may be potentially useful in treating resistant gliomas.

## Background

Gliomas comprise the majority of adult primary brain tumors diagnosed annually in the United States [1–4]. Gliomas are classified by the World Health Organization according to their morphologic characteristics into astrocytic, oligodendroglial, and mixed tumors [4, 5]. High grade gliomas (HGGs; grades III and IV) are the most common primary brain tumors in adults, and their malignant nature ranks them fourth in incidence of cancer death [2–4]. Approximately 15,000 patients die with glioblastomas (GBM, glioblastoma multiforme; a grade IV glioma) in the U.S.A. annually [1–4]. Malignant brain tumors kill approximately 140,000 people worldwide per year [6]. Standard treatment for GBM, which typically involves surgical resection followed by a combination of radiation and chemotherapy with the standard-of-care (SOC) temozolomide (TMZ), has not substantially improved overall survival (median survival remains 15 to 18 months, five-year survival rates are <10 %) [7, 8]. Prognosis is even poorer for recurrent disease, with response rates for cytotoxic chemotherapy typically in the range of 5 to 10 %, and 6-month progression-free survival rates of <15 % [9, 10].

One therapeutic strategy being actively pursued for multiple cancers is targeting angiogenesis, because without the ability to vascularize, a tumor cannot grow in size. Conversely, normal tissue is already vascularized and is not affected by angiogenic inhibition. Angiogenesis is greatly upregulated in HGGs compared to low-grade gliomas (LGGs) [8]. Angiogenesis is an essential process that provides excess nutrients to developing tumors even at a very early stage [11]. In fact, assessing angiogenesis is one of the most important criteria for grading tumors in patients [12]. In addition to cytotoxic chemotherapy, bevacizumab (Avastin®), an anti-VEGF antibody therapeutic, is also used to inhibit angiogenesis as a treatment for recurrent GBM, but it has not been found to significantly improve the clinical outcome [7, 8].

AG119 (previously referred to as 3⋅HCl [13]) is a small molecule discovered in our laboratory and found to possess anti-angiogenic (RTK inhibition) and antimicrotubule cytotoxic activity in a single molecule [13]. It was further found that AG119 possesses antitumor efficacy in two flank xenograft models – human MDA-MB-435 breast (free of melanoma cross-contamination [14]) and human U251 glioma, and anti-metastatic activity in a mouse orthotopic breast allograft (4 T1) model, with little systemic toxicity [13]. Due to the strong *in vivo* efficacy and potent angiogenic inhibitory activity of AG119 (decreased VEGFR2 (vascular endothelial growth factor receptor 2) and CD31/PECAM-1 immunohistochemical staining), and since we have previously shown that this compound is not a substrate for ATP binding cassette (ABC) transporters [13], a mechanism behind why many drugs fail to cross the blood–brain barrier [15], we tested AG119 as a potential anticancer therapy in an orthotopic allograft mouse (GL261) pre-clinical HGG model.

## Methods

### Orthotopic HGG model

Procedures for preclinical assessment of anti-cancer therapeutics in an orthotopic GL261 mouse glioma model were approved by the Institutional Animal Care and Use Committees (IACUC) at the Oklahoma Medical Research Foundation (OMRF) and the University of Oklahoma Health Sciences Center (OUHSC). Anesthetized C57BL6 mice (Harlan Laboratories) were implanted with GL261 mouse glioma cells (1 × 10^4^ cells in 6 μL PBS) (ATCC) as previously described for other glioma cells [16–18]. There were 5 treatment groups (untreated, anti-VEGF antibody, anti c-Met antibody, TMZ or AG119), which had 5–7 mice per group. Treatments were started once tumors were 10–20 mm^3^ as measured by MRI. Antibody therapies were administered at a dose of 1 mg/kg body weight i.v. via a tail-vein catheter every 3 days for up to 21 days [19]. TMZ and AG119 were administered at a dose of 30 mg/kg, i.p., twice weekly for 2 weeks. AG119 was dissolved in 5 % N-methylpyrrolidine (Pharmasolve; Sigma-Aldrich), 5 % solutol-15 (BASF, Bern, Switzerland) in sterile normal saline. TMZ was dissolved in 5 % DMSO and 5 % solutol-15 in sterile saline. Antibody therapies (anti-c-Met (Met (B-2): sc-8057; Santa Cruz Biotechnology Inc., Santa Cruz, CA) and anti-VEGF (anti-mouse VEGF-A; Biolegend Inc., San Diego, CA) were prepared in sterile saline. Control untreated tumor-bearing mice received the same solvent as for those that were treated with AG119 (vehicle control).

### MRI assessment of tumor volumes

MRI experiments were performed on a Bruker Bio-spec 7.0 Tesla/ 30-cm horizontal-bore magnet imaging system. Animals were immobilized with 1.5–2.5 % isoflurane and 0.8 L/min O_2_ and placed in a 72-mm quadrature volume coil for signal transmission, and a surface mouse-head coil was used for signal reception. Morphological T_1_ and T_2_-weighted MRI were used to assess tumor growth and calculate tumor volumes, as previously described [17, 18, 20], over a 25–35 day time period at 5–7 day intervals. Tumor volumes were calculated from multiple MRI slice datasets. Percent survival [Kaplan-Meier plots generated in Prism (GraphPad Software)] were also obtained from time-points when mice were euthanized 1–2 days prior to expected disease-initiated deaths. All animals were humanely euthanized (CO_2_ asphyxiation) when they met tumor burden criteria (tumors ≥ 150 mm^3^) and/or showed signs of illness, weight loss, poor body condition, porphyria, hypoactivity, restlessness, aggressiveness, ataxia, shallow, rapid and/or labored breathing, cachexia, failure to respond to stimuli, lack of inquisitiveness, vocalization, seizures, hunched posture and ruffled fur. Two animals died due to anesthesia complications, but were included in the survival data.

### Perfusion imaging (ASL)

Arterial spin label (ASL) perfusion images were obtained to calculate relative cerebral blood flow (rCBF) rates in tumors, as previously described [21]. Perfusion maps were obtained on a single axial slice of the brain located on the point of the rostro-caudal axis where the tumor had the largest cross-section [21]. The imaging geometry was a 3.5 × 3.5 mm^2^ slice, of 1.5 mm in thickness, with a single shot echo-planar encoding over a 64 × 64 matrix. An echo time of 20 ms and a repetition time of 18 s were used. To obtain perfusion contrast, the flow alternating inversion recovery scheme was used. Briefly, inversion recovery images were acquired using a slice-selective (SS) inversion of the same geometry as the imaging slice or a non-selective (NS) inversion slice concentric with the imaging slice.

Recovery curves obtained from each pixel of non-selective (*S*_*NS*_(*TI*) = *A* – *B* • *e*^-TI/T1*^) or selective (*S*_*SS*_(*TI*) = *A* – *B* • *e*^-TI/T1*^), with 1/*T*_1_* = 1/*T*_*1*_ + *CBF*/λ, inversion images were numerically fitted to derive the pixelwise *T*_*1*_ and *T*_1_* values, respectively [22]. The results were stored as maps for further analysis. These longitudinal recovery rates were then used to calculate the cerebral blood flow, *CBF* (ml/(100 g · min)) on a pixelwise basis using the following relationship: *CBF* = λ • [(1/*T*_1_*) – (1/*T*_*1*_)] [22]. The partition coefficient, λ, was scaled by assigning the generally adopted value of 0.9 ml/g [22]. Regions of interest (ROIs) were manually outlined around the tumor and a copy will be positioned onto the contralateral side of the brain for comparison purposes.

### Viability assay

U251 (TMZ-sensitive; low level of methyl guanine transferase (MGMT)) and T98G (TMZ-resistant; high level of MGMT) cells [23] (American Type Culture Collection, Manassas, VA) were maintained at Dulbecco’s minimal essential medium (DMEM) (Thermo Fisher Scientific) with 10 % Cosmic Calf Serum (CCS, Hyclone, Logan, UT) and added glutamine/pyruvate (HyClone) at 37 °C with 5 % CO_2_. Cells were treated with AG119 or TMZ (Sigma, St. Louis, MO) dissolved in Opti-MEM (Invitrogen, Carlsbad, CA) from 50 mM DMSO stock solutions. After 4 h of treatment, 10 % CCS was added and cells incubated for an additional 44 h. To determine viability, PrestoBlue (Invitrogen) was added as per manufacturer’s protocol and read on a microplate reader (BioTek, Winooski, VT). IC_50_ values were determined by nonlinear regression analysis in Prism 6.0 software (GraphPad, San Diego, CA).

### Statistical analyses

Statistical analyses were performed by using One-way ANOVA with a post Tukey’s multiple comparison test for evaluating differences in tumor volumes between untreated and treated groups. Data is represented as mean ± S.D., and p-values < 0.05 (*), < 0.01 (**), < 0.001 (***) were considered statistically significant. For survival curves, statistical differences were determined using a Log-rank (Mantel-Cox) test and a Gehan-Breslow-Wilcoxon test.

## Results

### Animal survival in different treatment groups

**Fig. 1:**
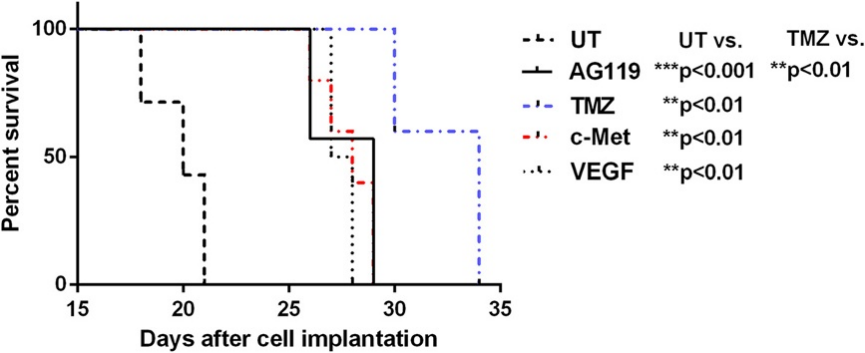
Survival data for treated and untreated GL261 glioma-bearing mice. AG119 (*n* = 7), TMZ (*n* = 5), anti-c-Met antibody (*n* = 5), and anti-VEGF antibody (*n* = 5) treated GL261 glioma-bearing mice, compared to untreated controls (UT) (*n* = 5). When comparing AG119 (***p < 0.001; *p* = 0.0003) or TMZ (**p < 0.01; *p* = 0.0016) to UT, there was a significant increase in survival

Percent animal survival were assessed for GL261-bearing mice that were either untreated or treated with either AG119, TMZ or antibody therapies against c-Met or VEGF. Percent survival of tumor-bearing mice treated with AG119 was significantly higher (*p* < 0.001) compared to untreated tumors, as depicted in Fig. 1. Compared to UT glioma-bearing mice, TMZ, anti-c-Met or anti-VEGF therapies all had significant increases in animal survival (*p* < 0.01 for each). It is important to note that TMZ, however, was found to have a significant increased percent survival when compared to AG119 (*p* < 0.01).

### Tumor volume determination from MR images

**Fig. 2:**
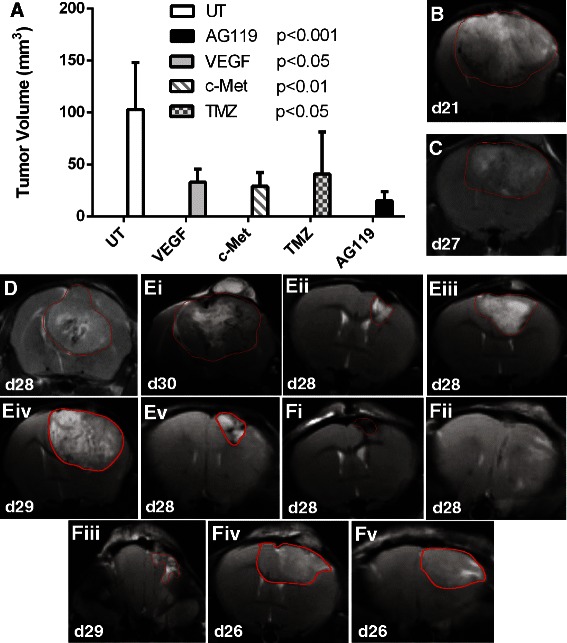
**a** Tumor volumes (mm^3^) of GL261 glioma-bearing animals either untreated (UT) or treated with anti-VEGF (*n* = 5) or anti-c-Met (*n* = 5) antibody therapies, TMZ (*n* = 5), or AG119 (*n* = 7), as measured by MRI. There was a significant decrease in tumors treated with either AG119 (***p < 0.001; *p* = 0.00067), TMZ (*p < 0.05; *p* = 0.03392), anti-VEGF (*p < 0.05; *p* = 0.01540) or ant-c-Met (**p < 0.01; *p* = 0.00539) when compared to untreated mice. **b**-**f** T_2_-weighted MR images of GL261 glioma-bearing mice. Tumors are outlined in red based on tumor boundary contrast with ‘normal’ brain tissue. **b** Representative untreated mouse (vehicle control) (21 days following GL261 cell implantation). **c** Anti-VEGF antibody treated mouse at 27 days after cell implantation. **d** Anti-c-Met antibody treated mouse at 28 days following cell implantation. **ei**-**v** TMZ-treated mice at 28–30 days following cell implantation. **fi**-**v** AG119-treated mice at 26–29 days following cell implantation. Mice were treated when tumor volumes were >10 mm^3^. Tumor volumes were measured by adding tumor areas in multiple 1 mm image slices. Each image is obtained from different mice in each treatment group

Morphological MRI was used to calculate tumor volumes. Tumor volumes (21–31 days following intracerebral implantation of GL261 cells) were found to be significantly lower for AG119-treated mice (*p* < 0.001) compared to untreated controls (Fig. 2a). Other current treatments for gliomas, which included anti-VEGF (*p* < 0.05) or anti-c-Met (*p* < 0.01) antibody therapies, or TMZ (*p* < 0.05), also had significant decreases in tumor volumes when compared to untreated tumors. Due to the large standard deviation for the TMZ tumor volumes, it was not possible to establish if there was a significant difference compared to AG119, as TMZ was found to significantly increase survival compared to AG119 (see Fig. 1). Representative MR images of untreated and treated mice are shown in Fig. 2b-f. AG119 compared well against other anti-glioma therapies including anti-VEGF and anti-c-Met antibody therapies, or TMZ, regarding tumor volumes.

### Tumor perfusion as a measure of tumor-associated vascular changes

**Fig. 3:**
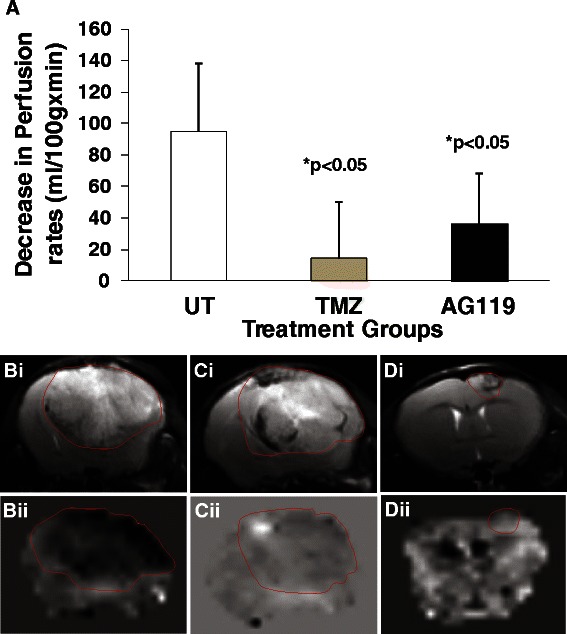
**a** Measured decreases in perfusion rates (ml/100 g x min) in untreated, TMZ- or AG119-treated GL261 mouse gliomas. Perfusion rates were measured using an arterial spin label perfusion MRI method, and relative decreases in tumors were obtained compared to the contralateral side. There were significant increases in tumor perfusion rates in both AG119 and TMZ-treated tumors (*p < 0.05), compared to untreated tumors, which had substantially decreased perfusion rates. **b**-**d** Perfusion MR images of untreated (**b**), TMZ (**c**), and AG119-treated (**d**) GL261 glioma-bearing mice. T2-weighted morphological images are in the top panels (**i**), and perfusion maps are in the bottom panels (**ii**). Dark region in panel Bii depicts decreased perfusion rates in an untreated tumor, which is not as severe in treated tumors (panels **cii** and **dii**). Tumors are outlined in red

In order to assess tumor microvasculature, tumor perfusion rates were obtained from MRI perfusion maps. Overall decreases in perfusion rates comparing tumor to contralateral regions were obtained. Perfusion data (Fig. 3a) indicates that both AG119 (see Fig. 3cii for an example) and TMZ (see Fig. 3dii for an example) were able to significantly increase perfusion rates (*p* < 0.05 for both; observed as reduced decreases in perfusion rates), compared to untreated tumors which normally have substantially decreased perfusion rates associated with an increased capillary bed and angiogenesis associated with a tumor (Fig. 3bii). Both AG119 (Fig. 3a, cii) and TMZ (Fig. 3a, dii) have a measurable anti-angiogenic effect in GL261 gliomas *in vivo,* which is keeping with the anti-angiogenic effects of TMZ in other orthotopic glioma models [24].

### Cytotoxic effect of AG119 on temozolomide (TMZ)-resistant cells

**Fig. 4:**
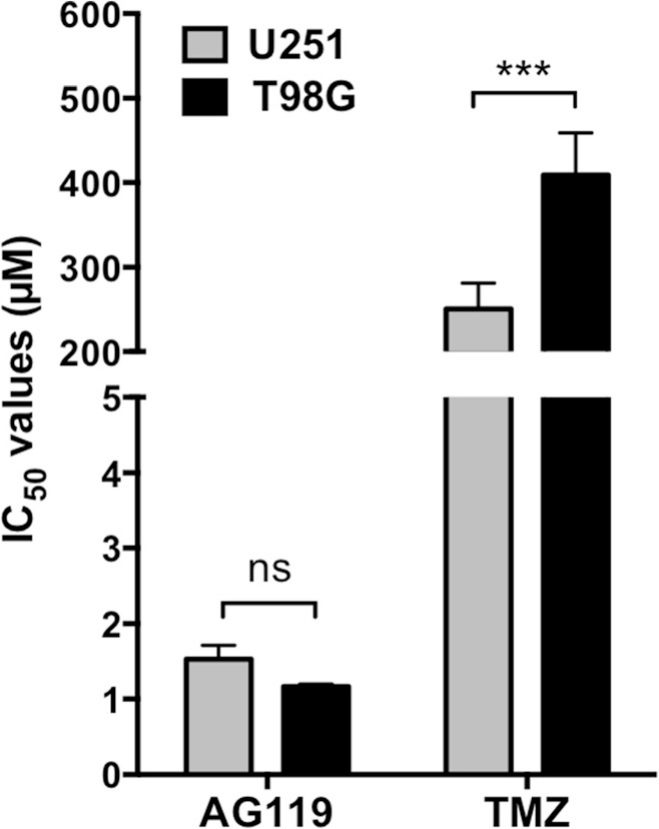
Cytotoxic effect of AG119 on temozolomide (TMZ)-resistant cells. Cells, U251 (TMZ-sensitive; MGMT^−^) and T98G (TMZ-resistant; MGMT^+^), were treated with AG119 or TMZ for 48 h and viability determined with Presto Blue. Data are mean IC_50_ values (μM) ± SEM, *n* = 4–9 independent experiments

Finally, it was determined whether AG119 retained sensitivity in a TMZ resistant glioma cell line. T98G cells overexpress O6-methylguanine-DNA-methyltransferase (MGMT), a DNA repair enzyme conferring resistance to a number of alkylating agents, including TMZ [25]. When comparing drug sensitivity of T98G cells to a relatively drug sensitive glioma line, U251, it was found that as expected the T98G cells were significantly less sensitive to TMZ (Fig. 4). It was also found that TMZ-resistant T98G cells were sensitive to AG119, as were the TMZ-sensitive U251 cells (Fig. 4; IC_50_ comparison).

## Discussion

It is well known that GL261 is a syngeneic mouse model of HHG in C57BL/6 mice with several morphological, pathological and genetic similarities to human GBM [26]. It has also been previously reported that the blood-tumor-barrier in GL261 gliomas are quite “leaky” which allows the penetration of various therapeutic compounds [27], as well as molecular targeting agents which we have reported on [28]. In this work we demonstrate in the GL261 glioma model that AG119, a small molecule with combined anti-angiogenic and antimicrotubule activity [13], results in a significant increase in animal percent survival (*p* < 0.001), as well as a significant decrease in GL261 HGG tumor growth *in vivo* (*p* < 0.001), compared to untreated tumors. AG119 was also found to be similar to the standard-of-care (SOC) TMZ, and relatively new anti-VEGF and anti-c-Met antibody targeted therapies. It should be noted that antibody therapies in this study were not optimized, i.e. doses used elicited therapeutic responses, but did not necessarily induce tumor regression [29]. Also of note, the survival data included anesthesia-related deaths for the AG119 treatment group which may not properly reflect the actual survival times for this treatment group, and should be repeated in future studies. Regardless, this proof-of-concept study does indicate that AG119 has anti-cancer activity in a pre-clinical glioma model.

AG119 was also found to significantly decrease tumor perfusion rates as well as TMZ (both *p* < 0.05, when compared to untreated tumors). Perfusion rates decrease in HGG, such as the GL261 model, as a result of the disorganized capillary architecture from vasculature associated with angiogenesis [22]. This is in keeping with previous findings that AG119 possessed inhibition of VEGFR2 kinase and anti-angiogenic activity in the chicken embryo chorioallantoic membrane (CAM) assay [13].

These data are encouraging because currently approved active antimicrotubule agents such as the taxanes do not readily cross the blood–brain barrier and are not useful for central nervous system (CNS) tumors, even though gliomas show taxane sensitivity in culture [30]. We have also previously shown that AG119 is not subject to resistance mechanisms common to other antimicrotubule agents (beta-III tubulin over-expression [25], P-glycoprotein) in tumors such as gliomas, suggesting that this agent may have a therapeutic advantage over current agents [13, 31]. This work suggests that AG119 is also not subject to MGMT mediated resistance, as is the case with TMZ. A study to further explore this idea would be a comparison of AG119 sensitivity in parental U251 cells as compared to T98G cells with MGMT knocked out. Thus AG119 may be useful in treating historically resistant gliomas.

## Conclusions

Taken together, in this study we demonstrate that AG119, a small molecule with combined anti-angiogenic and antimicrotubule activity, can significantly increase animal percent survival, significantly decrease GL261 HGG tumor growth, significantly decrease tumor vascularity, compared to untreated tumors, and suggests that this compound is also not subject to MGMT mediated resistance. These data support further exploration of the anticancer activity AG119 in HGG, perhaps together with SOC and/or newer agents like the c-Met inhibitors. In fact, it has been recently shown that combining a VEGF inhibitor (AG119 inhibits VEGFR2 kinase) with a c-Met inhibitor may have therapeutic advantage *in vivo* [32].

## Abbreviations

ABC: ATP binding cassette
ASL: Arterial spin label
CNS: Central nervous system
CBF: Cerebral blood flow
GBM: Glioblastomas
HGGs: High grade gliomas
LGGs: Low-grade gliomas
MRI: Magnetic resonance imaging
MGMT: Methyl guanine transferase
SOC: Standard-of-care
TMZ: Temozolomide
VEGF: Vascular endothelial growth factor
VEGFR2: Vascular endothelial growth factor receptor 2
